# Neutralization of interleukin-17 produced by gamma delta T cells constrains inflammation in experimental biliary atresia

**DOI:** 10.1186/2194-7791-2-S1-A21

**Published:** 2015-07-01

**Authors:** Christian Klemann, Arne Schröder, Anika Dreier, Nora Moehn, Stephanie Dippel, Thomas Winterberg, Anne Wilde, Yi Yu, Faikah Gueler, Anne Joerns, Eva Tolosa, Johannes Leonhardt, Jan D Haas, Immo Prinz, Claus Petersen, Joachim F Kuebler

**Affiliations:** Dept. of Pediatric Surgery, Hannover Medical School, Hannover, Germany; Dept. of Nephrology, Hannover Medical School, Hannover, Germany; Institute of Clinical Biochemistry, Hannover Medical School, Hannover, Germany; Department of Immunology, University Medical Center Hamburg-Eppendorf, Hamburg, Germany; Department of Pediatric Surgery, St. Bernward Hospital, Hildesheim, Germany; Institute of Immunology, Hannover Medical School, Hannover, Germany; MorphoSys AG, Martinsried/Planegg, Germany

## Meeting abstract

Biliary atresia (BA) is a rare disease of the infant with unknown pathogenesis. It is characterized by inflammatory, progressive destruction of the biliary system leading to liver fibrosis and progressive deterioration of liver function. Interleukin-17a (IL-17) has been identified as a cytokine driving inflammatory and autoimmune processes. We investigated the role of IL-17 and IL-17 producing cell populations in the pathogenesis of experimental and human BA.

In the rotavirus induced BA mouse model, symptomatic animals had a significantly increased hepatic transcription of IL-17. We identified gamma delta (γδ) T cells as the exclusive source of IL-17, while classical Th17 cells were completely absent. The increased number of IL-17^+^ γδ T cells in BA^+^ animals was associated with an up-regulation of typical markers of the IL-17-axis, such as IL17a, IL17f, RORγt, CCR6 and the IL-23-receptor. *In vivo*, blockage of IL-17 by administration of monoclonal antibodies ameliorated the clinical course of disease, improved survival and serum bilirubin, and reduced liver inflammation.

In human infants with BA, hepatic transcription of IL-17 was significantly up-regulated compared to patients with other neonatal cholestatic diseases, while no differences in IL-17 levels were detected in patient sera.

Taken together, IL-17 released by lymphocytes bearing the γδ T cell receptor appear to be a causative factor in the inflammatory destruction of the biliary system in experimental BA. Furthermore, our data suggest an important role of the IL-17 axis in human BA. Thus, targeting the IL-17 axis could be a promising approach for therapeutic interventions.

